# A two-way 224-Gbit/s PAM4-based fibre-FSO converged system

**DOI:** 10.1038/s41598-021-04315-3

**Published:** 2022-01-10

**Authors:** Hai-Han Lu, Chung-Yi Li, Wen-Shing Tsai, Poh-Suan Chang, Yan-Yu Lin, Yu-Ting Chen, Chen-Xuan Liu, Ting Ko

**Affiliations:** 1 Institute of Electro-Optical Engineering, National Taipei University of Technology, Taipei, 10608 Taiwan; 2 Department of Communication Engineering, National Taipei University, New Taipei City, 23741 Taiwan; 3 Department of Electrical Engineering, Ming Chi University of Technology, New Taipei City, 243303 Taiwan

**Keywords:** Engineering, Optics and photonics

## Abstract

A two-way 224-Gbit/s four-level pulse amplitude modulation (PAM4)-based fibre-free-space optical (FSO) converged system through a 25-km single-mode fibre (SMF) transport with 500-m free-space transmission is successfully constructed, which adopts injection-locked vertical-cavity surface-emitting lasers with polarisation-multiplexing mechanism for a demonstration. Compared with one-way transmission, two-way transmission is an attractive architecture for fibre-FSO converged system. Two-way transmission over SMF transport with free-space transmission not only reduces the required number of fibres and the setups of free-space transmission, but also provides the advantage of capacity doubling. Incorporating dual-wavelength four-level pulse amplitude modulation (PAM4) modulation with polarisation-multiplexing mechanism, the transmission capacity of fibre-FSO converged system is significantly enhanced to 224 Gbit/s (56 Gbit/s PAM4/wavelength × 2-wavelength × 2-polarisation) for downlink/uplink transmission. Bit error rate and PAM4 eye diagrams (downstream/upstream) perform well over 25-km SMF transport with 500-m free-space transmission. This proposed two-way fibre-FSO converged system is a prominent one not only because of its development in the integration of fibre backbone with optical wireless extension, but also because of its advantage in two-way transmission for affording high downlink/uplink data rate with good transmission performance.

## Introduction

It is vitally important to provide broadband integrated services with long-haul transmission and high transmission capacity. Single-mode fibre (SMF) affords good transmission performance with low dispersion and low attenuation. Noticeable advantages are afforded by implementing the SMF to enhance the coverage area at relatively high-speed using the characteristics of optical fibre. Free-space optical (FSO) communications, which transport optical signals by laser light propagation in free-space, have obtained extensive attention for practically solving the problem of short-reach RF wireless communications^1–4^. Given the developments in SMF and FSO technologies, fibre-FSO converged system is expected to provide hundreds of gigabit services through the long-distance fibre transmission with long-reach FSO communication. To meet the targets of long-haul transmission and high transmission capacity, a two-way 224-Gbit/s four-level pulse amplitude modulation (PAM4) fibre-FSO converged system through 25-km SMF transport with 500-m free-space transmission is thereby established, adopting injection-locked vertical-cavity surface-emitting lasers (VCSELs) with polarisation-multiplexing mechanism. Two injection-locked VCSELs are orthogonally polarized and multiplexed with a wavelength spacing of 8 nm. The transmission rate of two-way fibre-FSO converged system is significantly enhanced by integrating PAM4 modulation and injection-locked VCSELs with polarisation-multiplexing mechanism. Due to the enhancement ability of VCSEL’s resonance frequency, injection locking technique effectively improves the modulation response of a PAM4-based fibre-FSO converged system^5,6^. PAM4 modulation is an effective optical mechanism for increasing the transmission rate of fibre-FSO converged system. Polarisation-multiplexing is another effective optical mechanism for enhancing the transmission rate of fibre-FSO converged system. As each wavelength is transported with orthogonal polarisation, the utilization of a polarisation-multiplexing mechanism doubles the transmission rate^7–10^. Combining PAM4 modulation with polarisation-multiplexing mechanism guides a way to considerably increase the transmission rate of the fibre-FSO converged system. Compared with non-return-to-zero modulation with single-wavelength and single-polarisation state, dual-wavelength PAM4 modulation with polarisation-multiplexing mechanism yields up to eight times [2 (dual-wavelength) × 2 (PAM4 modulation) × 2 (polarisation-multiplexing)] total transmission rate.

Creating a simple polarisation demultiplexing mechanism is critically important for a polarisation-multiplexing fibre-FSO converged system. Uniting a tunable optical band-pass filter (TOBPF) with a polarisation beam splitter (PBS) is an easy and effective polarisation demultiplexing mechanism. It is attractive because it prevents the requirement for a complicated polarisation demultiplexing mechanism^11,12^. Employing a TOBPF with a PBS, the orthogonally polarized wavelengths are dramatically filtered and demultiplexed because they are located at different wavelengths and polarised with orthogonal polarisations.

Yao et al. achieved an orthogonal frequency-division multiplexing (OFDM)-based fibre-FSO converged system with heterodyne detection to increase the receiving sensitivity of FSO communication^13^. However, the high peak-to-average power ratio is a critical concern for an OFDM-based fibre-FSO converged system. PAM4 modulation is better than OFDM modulation because of its low power feature^14^. Moreover, the 1 m free-space transmission distance is much smaller than the corresponding value of 500 m adopted in this demonstrated fibre-FSO converged system. Additionally, costly external modulators and sophisticated heterodyne detection technique are required to build such expensive and complex OFDM-based fibre-FSO converged system. Transmitters with direct modulation are attractive for fibre-FSO converged system because they cost less and design easier than transmitters with external modulation. Our former study demonstrated an 800-Gbit/s wavelength-division-multiplexing PAM4 FSO communication with spatial light modulator-based beam tracking technology over 200 m free-space link^15^. The 200 m free-space transmission distance is significantly less than the associated value of 25.5 km (25-km SMF + 500-m free-space) developed in our proposed converged system. M. Singh et al. presented a 40-Gbit/s FSO transmission based on orbital angular momentum multiplexed beams^16^. Moreover, a radio over FSO link under harsh weather conditions employing mode-division multiplexing was presented^17^. Furthermore, a high-speed radio over FSO transmission link under dust environment conditions employing hybrid wavelength- and mode- division multiplexing was demonstrated^18^. However, the aggregate transmission rates of 40 Gbit/s, 40 Gbit/s, and 160 Gbit/s and are less than the corresponding value of 224 Gbit/s operated in this demonstration. Besides, for a real implementation, building a two-way transmission rather than a one-way transmission is important. A one-way lightwave transmission system has a lot of room for improvement when considering a two-way transmission. Therefore, a two-way fibre-FSO converged system for delivering symmetric downlink/uplink data rate is constructed to meet the target. In this study, a two-way 224-Gbit/s PAM4-based fibre-FSO converged system over 25-km SMF transport with 500-m free-space transmission is constructed, which adopts directly modulated VCSELs with polarisation-multiplexing mechanism and injection-locked technique as a presentation. To the best of our knowledge, this system is the first to practically construct a two-way PAM4-based fibre-FSO converged system with a high downlink/uplink transmission rate of 224 Gbit/s. Since that both downlink and uplink PAM4 data signals are transmitted by the same SMF, the Rayleigh backscattering (RB) noise is considered in this two-way PAM4-based fibre-FSO converged system^19–21^. Additionally, the link availability of FSO communications greatly relies on weather conditions which influence the signal intensity^22,23^. Thus, atmospheric turbulence because of heavy rain is concerned in a 500-m free-space transmission. With a thorough examination, impressively low bit error rate (BER) of 10^−9^ and qualified PAM4 eye diagrams are acquired in this constructed two-way PAM4-based fibre-FSO converged system. Results show that such proposed two-way 224-Gbit/s PAM4-based fibre-FSO converged system is advantageous for developing a two-way long-haul fibre communication with free-space transmission. Moreover, it can be used in actual commercial systems to provide communication links in specific areas where RF communications are prohibited, such as in hospitals, in cabins or in oil refineries. It affords a secure link, rather than a fibre-wireless converged system, because the laser beam does not interfere with the RF signals. For future extension, a two-way fibre-FSO converged system with ultra-long-haul transmission and ultra-high transmission capacity is expected to be built by adopting PAM4 modulation and multi-stage injection-locked VCSELs^24^ with polarisation-multiplexing mechanism.

## Results

### The optical spectra of VCSEL1 and the modulation responses of VCSELs in free-running and injection-locked states with 56 Gbit/s PAM4 data signal

**Figure 1:**
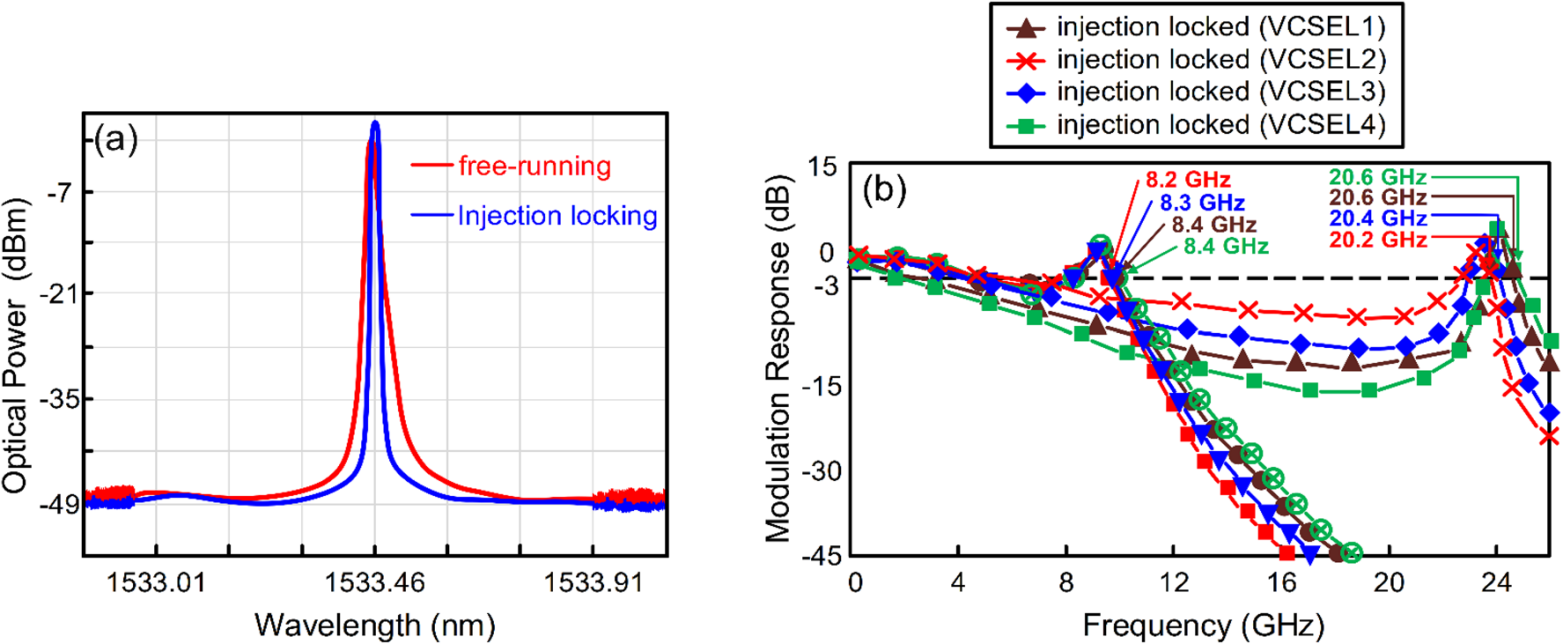
(**a**) The optical spectra of VCSEL1 in free-running and injection-locked states with 56 Gbit/s PAM4 data signal. (**b**) The modulation responses of VCSELs in free-running and injection-locked scenarios with 56 Gbit/s PAM4 data signal.

Figure 1a shows the optical spectra of VCSEL1 in free-running and injection-locked states with 56 Gbit/s PAM4 data signal. It is to be observed that as the VCSEL1 (1533.44 nm) is injection-locked, its optical spectrum shifts to a somewhat longer wavelength (1533.46 nm). With injection locking, the optical spectrum of the injection-locked laser moves to a slightly longer wavelength, instead of its original free-running wavelength. Injection locking behavior happens as a slave laser (VCSEL1) with a wavelength that is a slightly shorter than that of the master laser [distributed feedback (DFB) laser diode1 (LD1)]. In addition, it is to be found that the injection-locked VCSEL1’s peak power is enhanced and the injection-locked VCSEL1’s linewidth is suppressed. An injection-locked technique enhances the intensity and suppresses the linewidth of the injection-locked laser^25,26^. Four directly modulated DFB LDs could be used to replace four VCSELs with injection locking. Directly modulated DFB LD, however, has a large frequency chirp which degrades the transmission performance. An effective approach to reduce the frequency chirp is to operate laser with suppressed linewidth. The linewidth will be suppressed as the modulated VCSEL is injection-locked and leads to better transmission performance. Besides, the modulation responses of VCSELs in free-running and injection-locked scenarios with 56 Gbit/s PAM4 data signal are shown in Fig. 1b. With injection locking, the 3-dB modulation response is considerably enhanced from 8.4 (VCSEL1)/8.2 (VCSEL2)/8.3 (VCSEL3)/8.4 (VCSEL4) GHz to 20.6 (VCSEL1)/20.2 (VCSEL2)/20.4 (VCSEL3)/20.6 (VCSEL4) GHz. With injection locking, the difference between the injection-locked laser mode and the cavity mode is obtained, and thus produces a considerable resonance frequency improvement and a significant 3-dB modulation response enhancement^24^. Provided that the transmission rate is $\sqrt 2$ times the bandwidth, a PAM4-based fibre-FSO converged system with a data rate of 224 Gbit/s [20.2 × $\sqrt 2$ × 2 (PAM4 modulation) × 2 (dual-wavelength) × 2 (polarisation-multiplexing mechanism) > 224] in total can be practically constructed.

### BER performances of 224 Gbit/s PAM4-based fibre-FSO converged system (downlink transmission) under different scenarios and eye diagrams of 56 Gbit/s PAM4 signal in different states

**Figure 2:**
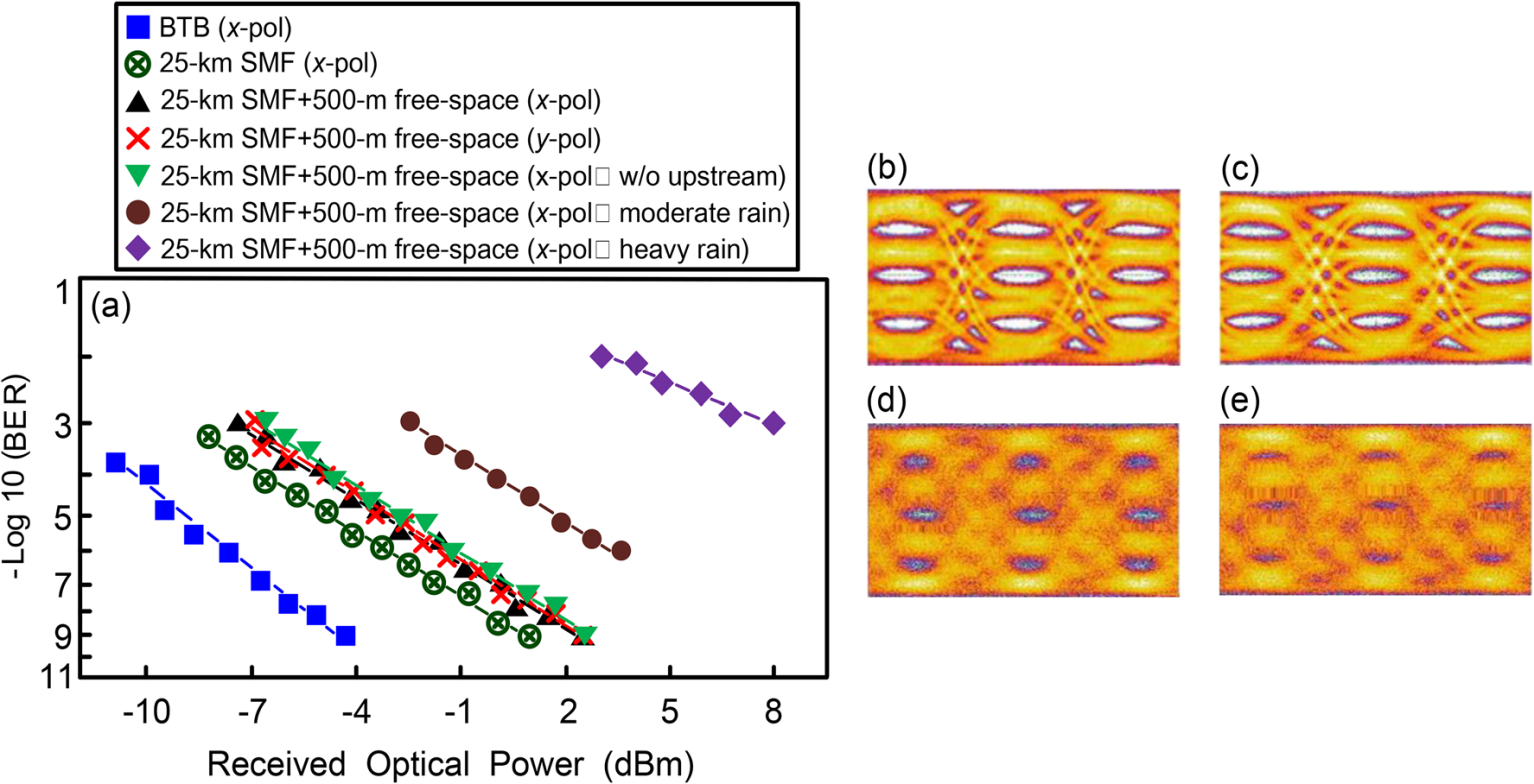
(**a**) The BER performances of 224 Gbit/s PAM4-based fibre-FSO converged system (downlink transmission) under different scenarios of back-to-back (BTB, *x*-polarisation), over 25-km SMF transport (*x*-polarisation; sunny weather), over 25-km SMF transport with 500-m free-space transmission (*x*- and *y*-polarisations; sunny weather), and over 25-km SMF transport with 500-m free-space transmission (*x*-polarisation; moderate/heavy rain). The eye diagrams of 56 Gbit/s PAM4 signal in (**b**) BTB state (*x*-polarisation) and that over 25-km SMF transport with 500-m free-space transmission (*x*-polarisation) under (**c**) sunny weather, (**d**) moderate rain, and (**e**) heavy rain conditions.

Implementing a 224-Gbit/s/25.5-km (25-km SMF + 500-m free-space) PAM4-based fibre-FSO converged system is quite challenging, especially for acquiring good BER performance and competent PAM4 eye diagrams. Figure 2a presents the BER performances of 224 Gbit/s PAM4-based fibre-FSO converged system (downlink transmission) under different scenarios of back-to-back (BTB, *x*-polarisation), over 25-km SMF transport (*x*-polarisation; sunny weather), over 25-km SMF transport with 500-m free-space transmission (*x*- and *y*-polarisations; sunny weather), and over 25-km SMF transport with 500-m free-space transmission (*x*-polarisation; moderate/heavy rain). Under a 10^−9^ BER operation, a 5.6-dB power penalty appears between the BTB scenario and that over 25-km SMF transport (*x*-polarisation; sunny weather). The 5.6 dB power penalty primarily arises from the fibre dispersion-induced penalty due to 25-km SMF transport. Moreover, when the BER is 10^−9^, a 1.1-dB power penalty emerges between the 25-km SMF scenario (*x*-polarisation; sunny weather) and that over 25-km SMF transport with 500-m free-space transmission (*x*- or *y*-polarisation; sunny weather). Such a 1.1-dB power penalty chiefly results from the atmospheric attenuation because of 500 m free-space transmission and coupling loss for coupling laser light on the fibre ferrule^27,28^. Through 500-m free-space transmission, laser light must be fitted into the fibre ferrule to avoid large coupling loss. Furthermore, it is to be observed that the BER performances of *x*-polarisation (with/without uplink PAM4 signals) and *y*-polarisation (with uplink PAM4 signals) are nearly the same. These show that there is no correlation between BER performance and polarisation state, and there is no correlation between downlink and uplink transmissions due to 4 nm coarse wavelength spacing between the downstream/upstream adjacent wavelengths. Additionally, in moderate rain, the BER increases to a 10^−6^ order of magnitude due to atmospheric attenuation from the moderate rain. In heavy rain, the BER performance severely degrades to a 10^−3^ order of magnitude due to high atmospheric attenuation from the heavy rain. The performance of FSO communication is highly dependent of the atmospheric channel condition. When the atmospheric channel condition is poor, then a transmitted laser light is significantly influenced by atmospheric turbulence. The inhomogeneity in the temperature and pressure over the atmospheric channel changes the refractive index of the atmosphere and brings on atmospheric attenuation^29–31^. Through 500 m free-space transmission, the atmospheric attenuation coefficient changes from 0.0042 dB/m (sunny weather) to 0.025 dB/m (heavy rain). The atmospheric attenuation coefficient (σ) can be expressed as^31^:

1 $$\sigma = \frac{3.91}{V}\left( {\frac{\lambda }{550 \;nm}} \right)^{ - q}$$

where *V* is the visibility, *λ* is the wavelength, and *q* is the size distribution of the scattering particles. In sunny weather, a 500-m free-space transmission provides high link accessibility because of slight atmospheric attenuation. In heavy rain, a 500-m free-space transmission provides low link accessibility because of heavy atmospheric attenuation. In extremely heavy rain, however, a 500-m free-space transmission will be interrupted due to extremely high atmospheric attenuation coefficient of 0.061 dB/m.

Figure 2b–e exhibit the eye diagrams of 56 Gbit/s PAM4 signal in BTB state (*x*-polarisation) and that over 25-km SMF transport with 500-m free-space transmission (*x*-polarisation) under different weather conditions. In BTB sate, open eye diagrams are attained with 10^−9^ BER operation (Fig. 2b). In the state over 25-km SMF transport with 500-m free-space transmission (sunny weather), a clear eye pattern is observed with 10^−9^ BER operation (Fig. 2c). Over 25-km SMF transport with 500-m free-space transmission (moderate rain), the eye diagrams are blurred with 10^−6^ BER operation (Fig. 2d). Over 25-km SMF transport with 500-m free-space transmission (heavy rain), however, the eye diagrams are closed with 10^−3^ BER operation (Fig. 2e).

### BER performances of 224 Gbit/s PAM4-based fibre-FSO converged system (uplink transmission) in sunny weather under different scenarios and eye diagrams of 56 Gbit/s PAM4 signal in different conditions

**Figure 3:**
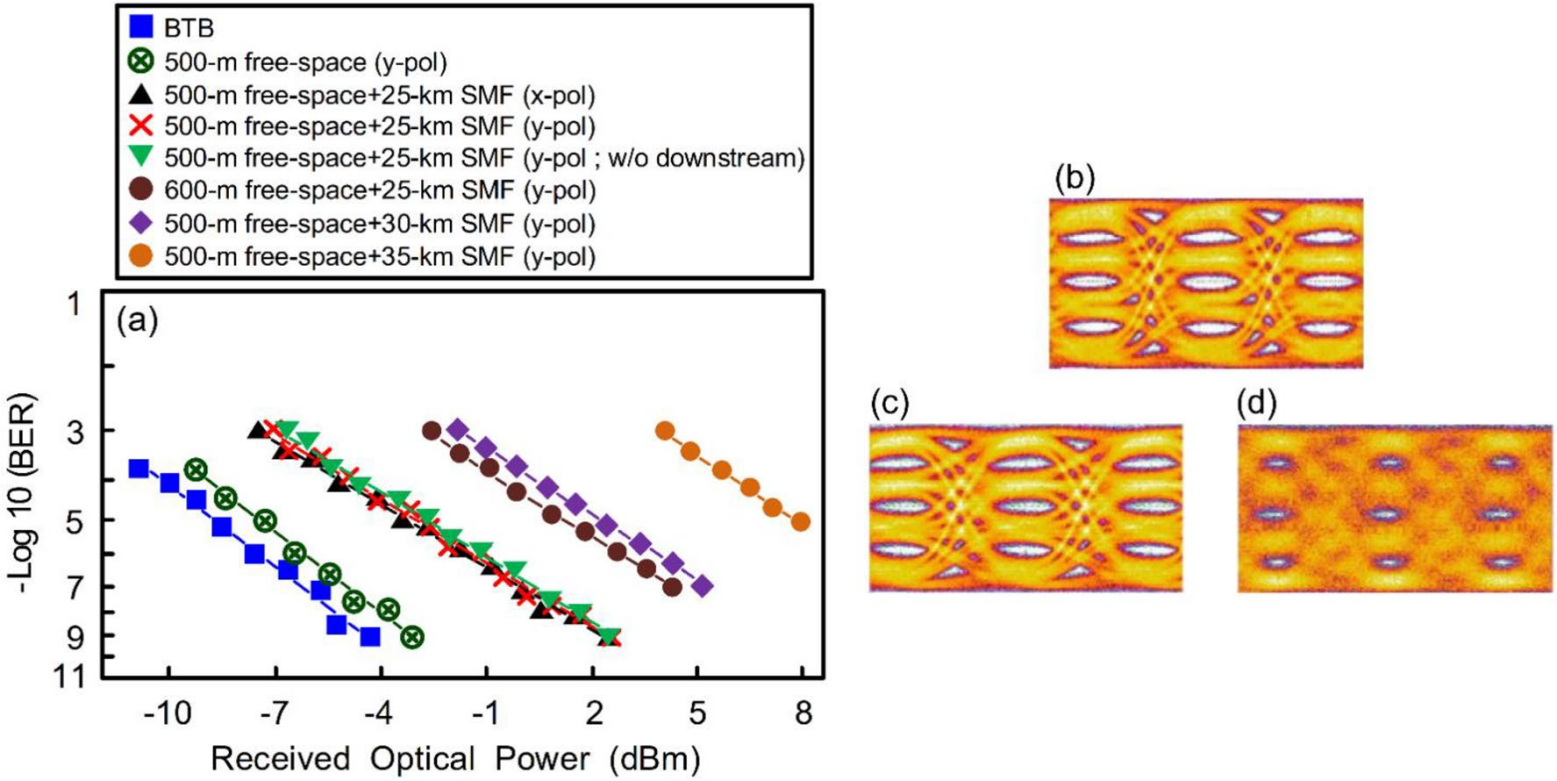
(**a**) The BER performances of 256 Gbit/s PAM4-based fibre-FSO converged system (uplink transmission) in sunny weather under the scenarios of BTB (*y*-polarisation), over 500-m free-space transmission (*y*-polarisation), over 500-m free-space and 25-km SMF transmissions (*x*- and *y*-polarisations), over 600-m free-space and 25-km SMF transmissions (*y*-polarisation), over 500-m free-space and 30-km SMF transmissions (*y*-polarisation), as well as over 500-m free-space and 35-km SMF transmissions (*y*-polarisation). The eye diagrams of 56 Gbit/s PAM4 signal under the conditions (**b**) over 500-m free-space transmission (*y*-polarisation), (**c**) over 500-m free-space and 25-km SMF transmissions (*y*-polarisation), and (d) over 600-m free-space and 25-km SMF transmissions (*y*-polarisation).

Figure 3a exhibits the BER performances of 224 Gbit/s PAM4-based fibre-FSO converged system (uplink transmission) in sunny weather under the scenarios of BTB (*y*-polarisation), over 500-m free-space transmission (*y*-polarisation), over 500-m free-space and 25-km SMF transmissions (*x*- and *y*-polarisations), over 600-m free-space and 25-km SMF transmissions (*y*-polarisation), over 500-m free-space and 30-km SMF transmissions (*y*-polarisation), as well as over 500-m free-space and 35-km SMF transmissions (*y*-polarisation). When the BER is 10^−9^, there exists 1.1 dB power penalty between the scenarios of BTB (*y*-polarisation) and 500-m free-space transmission (*y*-polarisation). This 1.1 dB power penalty is due to the atmospheric attenuation caused by 500-m free-space transmission and coupling loss for doublet lens 1 coupling laser beam on the fibre ferrule. And further, as the BER is 10^−9^, there exists 5.7 dB power penalty between the scenarios of 500-m free-space transmission (*y*-polarisation) and 500-m free-space with 25-km SMF transmission (*x*- and *y*-polarisations). Such 5.7 dB power penalty is mainly due to the fibre dispersion because of 25-km SMF transport. Additionally, it is to be found that the BER performances of *x*-polarisation (with downlink PAM4 signals) and *y*-polarisation (with/without downlink PAM4 signals) are almost the same. Furthermore, to have more associations with the free-space transmission distance, SMF length, and BER performance, we extend the free-space transmission distance and SMF length respectively to evaluate the BER performance. As the free-space transmission attains 600-m (600-m free-space + 25-km SMF), BER increases to 10^−7^ due to more atmospheric attenuation and larger coupling loss. As the laser is delivered over 600-m free-space transmission, it is difficult to completely concentrate the laser beam on the fibre ferrule due to laser’s natural expansion, resulting in a large coupling loss. In addition, as the SMF length increases to 30 km (500-m free-space + 30-km SMF), BER performance degrades to a 10^−7^ order of magnitude due to more RB noise. As the SMF length further increases to 35 km (500-m free-space + 35-km SMF), BER performance severely degrades to a 10^−5^ order of magnitude due to higher RB noise. Because both downstream and upstream 256 Gbit/s PAM4 data signals are transmitted by the same SMF length, the RB noise thereby restricts the downstream and upstream transmission performances. When the SMF length is less than 25 km, lower RB noise leads to better BER performance. Whereas as the SMF length exceeds 25 km, higher RB noise results in poorer BER performance^19–21^. In long-haul lightwave transport systems (> 40 km), the impairment is dominated by fiber dispersion-induced distortion. Given that the maximum SMF length in this demonstration is only 35 km (< 40 km), the impairment is thereby dominated by RB noise rather than fiber dispersion-induced distortion. Regarding eye diagrams, an open PAM4 eye diagram is attained (Fig. 3b) under the condition through 500-m free-space transmission (*y*-polarisation). A clear PAM4 eye diagram is obtained (Fig. 3c) under the condition through 500-m free-space and 25-km SMF transmissions (*y*-polarisation). Whereas a blurred PAM4 eye diagram is acquired (Fig. 3d) under the condition through 600-m free-space and 25-km SMF transmissions (*y*-polarisation).

## Discussion

**Figure 4:**
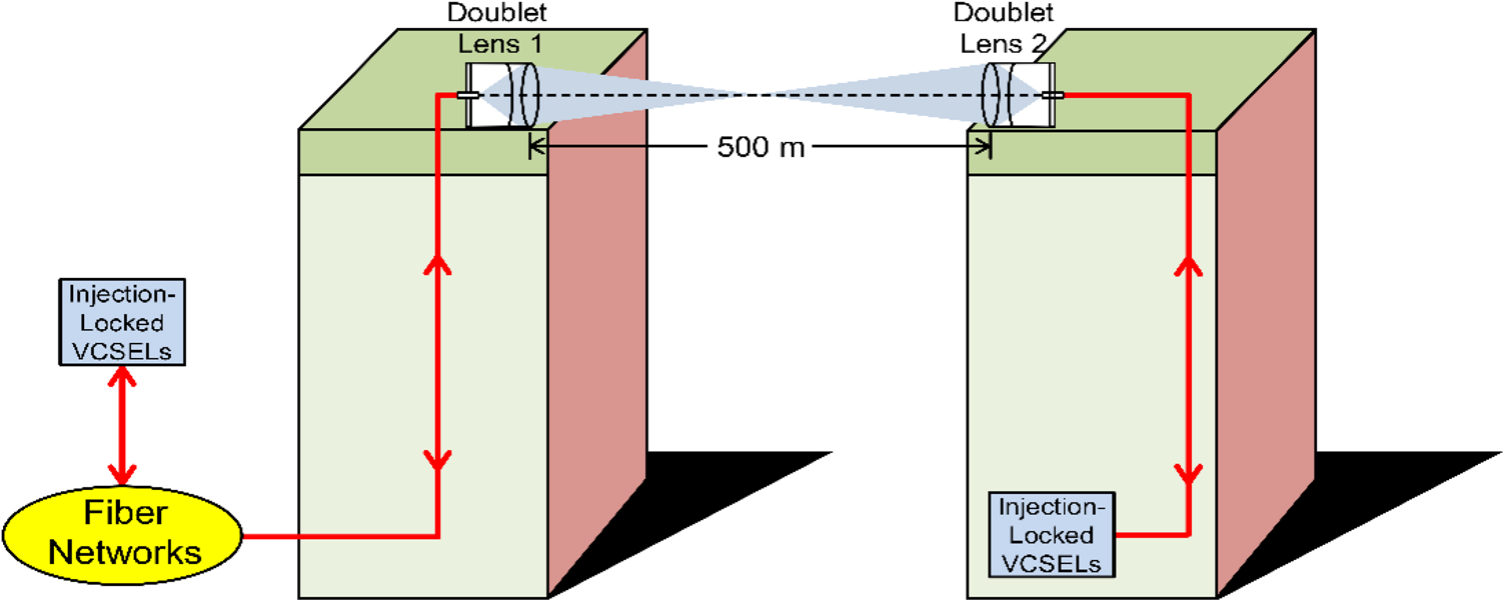
The layout of the doublet lenses-based two-way fibre-FSO converged system.

The layout of the doublet lenses-based two-way fibre-FSO converged system is presented in Fig. 4. Doublet lens emits and receives optical signals wirelessly to integrate fibre networks and FSO communications. Two doublet lenses are placed on the rooftops of two mansions to transmit/receive lasers through free-space in a two-way communication. Doublet lens is composed of one concave lens and one convex lens, with focal length and diameter of 150 and 50.8 mm. Since the numerical aperture of the optical fiber is 0.14, the laser light’s diameter (*d*) can be calculated as 42 mm [2 × (150 × 0.14) = 42]. The doublet lens 1’s diameter (50.8 mm) is larger than laser light’s diameter (42 mm) to make an FSO communication workable. The corresponding beam radius (*r*) is given by

2 $$r = \frac{2.3}{{SFC \times 2\pi }} = 3.6 \;(\mu m{)}$$

where *SFC* is the spatial frequency cutoff. Thus, the laser light’s divergent angle ($\theta$) can be calculated as:

3 $$\theta = \frac{3.6\; (\mu m)}{{150 \;(mm)}} = 2.4 \times 10^{ - 5}$$

Over an *L*-m free-space link, the laser light’s diameter (*d*_*L*_) must be smaller than doublet lens 2’s diameter (*d*_*L*_ < 50.8 mm) to avoid large coupling loss and collect more laser light:

4 $$d_{L} = \sqrt {d^{2} + \left( {2\theta L} \right)^{2} } = \sqrt {42^{2} + \left( {0.048L} \right)^{2} } < 50.8$$

*L* is obtained as 595.4 m, indicating the maximum free-space link is 595.4 m. The free-space link in this demonstration is 500 m (< 595.4 m), which meets the free-space link demand.

## Methods

### Framework of the demonstrated two-way 224-Gbit/s PAM4-based fibre-FSO converged system adopting injection-locked VCSELs with polarisation-multiplexing mechanism over 25-km SMF transport with 500-m free-space transmission

**Figure 5:**
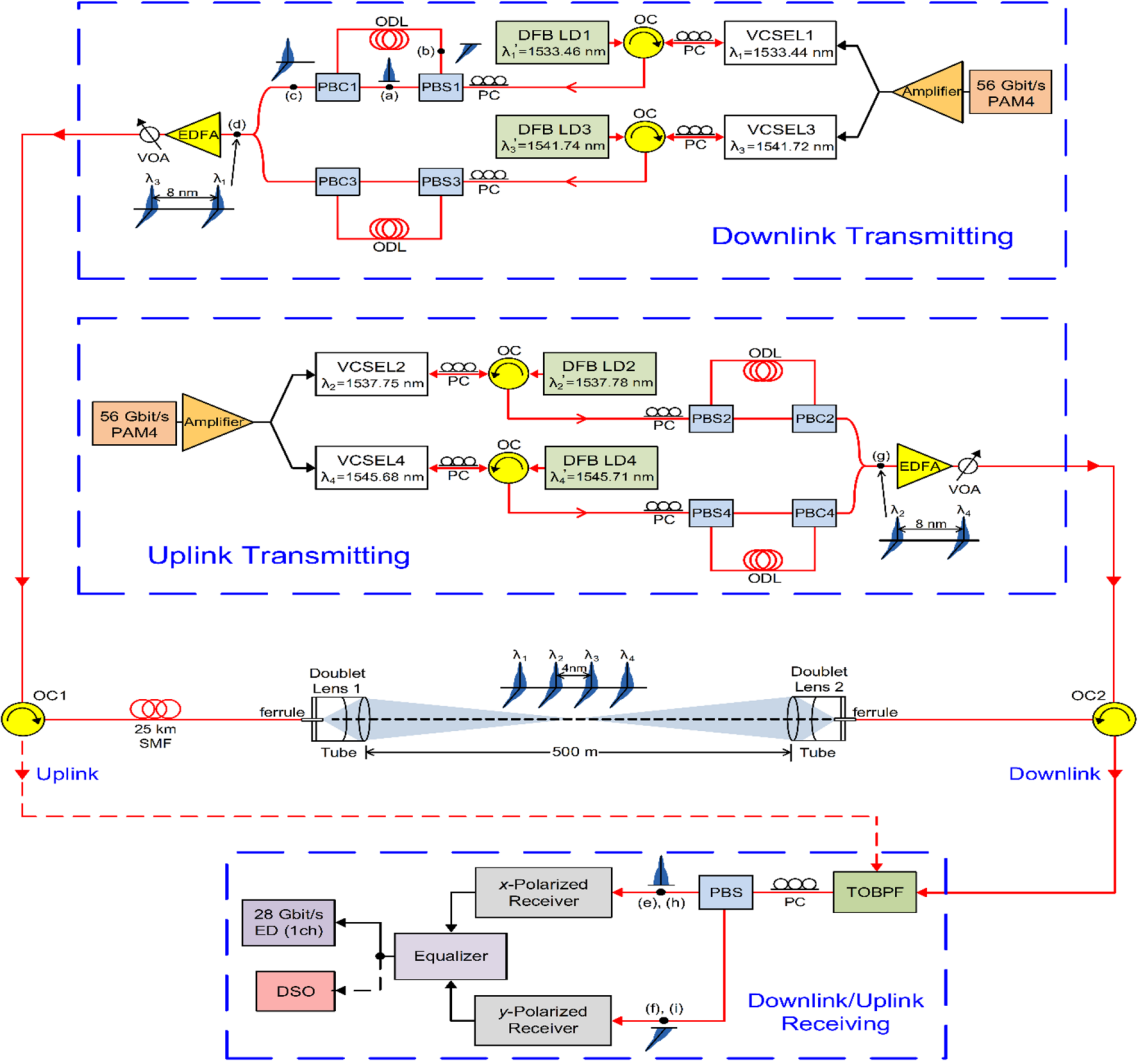
Framework of the demonstrated two-way 224-Gbit/s PAM4-based fibre-FSO converged system adopting injection-locked VCSELs with polarisation-multiplexing mechanism.

The framework of the demonstrated two-way 224-Gbit/s PAM4-based fibre-FSO converged system adopting injection-locked VCSELs with polarisation-multiplexing mechanism over 25-km SMF transport with 500-m free-space transmission is exhibited in Fig. 5. For downlink transmission, the 56 Gbit/s PAM4 signal created from the PAM4 signal generator is enhanced by a wideband amplifier, split by a 1 × 2 RF splitter, and inputted into VCSEL1 and VCSEL3. The light emitted from a DFB LD injects into a VCSEL (DFB LD1/1533.46 nm → VCSEL1/1533.44 nm, DFB LD3/1541.74 nm → VCSEL3/1541.72 nm) by utilizing an optical circulator (OC) with a polarisation controller (PC). After that, the 56 Gbit/s optical PAM4 signal is separated into *x*- [inset (a)] and *y*-polarized [inset (b)] signals by a PC with a PBS. Then, these two polarized optical signals are combined by a polarisation beam combiner (PBC) [inset (c)]. If two polarisations are not separated by a PBS and then not combined by a PBC, an optical PAM4 signal with two orthogonal polarisations cannot be obtained. Given that the group velocities of *x*- and *y*-polarized signals are different, one of the optical paths deploys an optical delay line to make up for the phase (optical path) difference between the two polarized signals. Two optical PAM4 signals with *x*- and *y*-polarisations are coupled by a 2 × 1 optical coupler [inset (d)], promoted by an erbium-doped fibre amplifier, adjusted by a variable optical attenuator (VOA), and then delivered over 25-km SMF and 500-m free-space by two OCs (OC1 and OC2). Two doublet lenses at the transmitting/receiving site are utilized to implement a 500-m free-space transmission. Afterward, two integrated optical PAM4 signals with *x*- and *y*-polarisations go through an OC2 and a TOBPF with 0.56 nm 3-dB bandwidth, and deliver through a PC with a PBS to separate *x*- and *y*-polarized optical PAM4 signals. Then, the *x*-polarized carrier with a 56-Gbit/s optical PAM4 signal [inset (e)] is sent to an *x*-polarized receiver. After electrical equalization, the PAM4 signal is calculated by BER parameter using a 28-Gbit/s error detector (ED). And further, the 56 Gbit/s PAM4 signal’s eye diagrams are caught using a digital storage oscilloscope (DSO). Similarly, the *y*-polarized carrier with a 56-Gbit/s optical PAM4 signal [inset (f)] is supplied in a *y*-polarized receiver. After equalization, the PAM4 signal undergoes a real-time BER measurement using an ED. Moreover, a DSO is used to catch the eye diagrams.

For uplink transmission, the 56 Gbit/s PAM4 signal is driven via a broadband amplifier, divided by a 1 × 2 power divider, and supplied to VCSEL2 and VCSEL4. The light emitted from a DFB LD is injected into a VCSEL (DFB LD2/1537.78 nm → VCSEL2/1537.75 nm, DFB LD4/1545.71 nm → VCSEL4/1545.68 nm) through an OC integrated with a PC. Successively, the 56 Gbit/s optical PAM4 signal is divided into two parts with orthogonal states using a PC with a PBS. To offset the phase difference between the two optical paths, one of the optical paths deploys an optical delay line. Two optical PAM4 signals with *x*- and *y*-polarisations are coupled by an optical coupler [inset (g)], magnified by an optical amplifier, controlled by a VOA, and communicated over 500-m free-space and 25-km SMF by two OCs (OC2 and OC1). Next, two integrated optical PAM4 signals are circulated by an OC1, passed through a TOBPF, and delivered through a PC with a PBS to split *x*- and *y*-polarized optical PAM4 signals. Next, the *x*-polarized/*y*-polarized carrier with a 56-Gbit/s optical PAM4 signal [inset (h)/inset (i)] is inputted into an *x*-polarized/*y*-polarized receiver. After equalization, the performance of PAM4 signal is analyzed by BER parameter using a 28-Gbit/s ED. In addition, the 56 Gbit/s PAM4 signal’s eye diagrams are taken by a DSO.
